# A sampling strategy for longitudinal and cross-sectional analyses using a large national claims database

**DOI:** 10.3389/fpubh.2024.1257163

**Published:** 2024-02-01

**Authors:** Timothy L. McMurry, Jennifer M. Lobo, Soyoun Kim, Hyojung Kang, Min-Woong Sohn

**Affiliations:** ^1^Department of Public Health Sciences, University of Virginia, Charlottesville, VA, United States; ^2^Department of Social Welfare, Ewha Womans University, Seoul, Republic of Korea; ^3^Department of Kinesiology and Community Health, University of Illinois, Champaign, IL, United States; ^4^Department of Health Management and Policy, University of Kentucky, Lexington, KY, United States

**Keywords:** diabetes, Medicare claims, sample, longitudinal analysis, cross-sectional design

## Abstract

**Importance:**

The United States (US) Medicare claims files are valuable sources of national healthcare utilization data with over 45 million beneficiaries each year. Due to their massive sizes and costs involved in obtaining the data, a method of randomly drawing a representative sample for retrospective cohort studies with multi-year follow-up is not well-documented.

**Objective:**

To present a method to construct longitudinal patient samples from Medicare claims files that are representative of Medicare populations each year.

**Design:**

Retrospective cohort and cross-sectional designs.

**Participants:**

US Medicare beneficiaries with diabetes over a 10-year period.

**Methods:**

Medicare Master Beneficiary Summary Files were used to identify eligible patients for each year in over a 10-year period. We targeted a sample of ~900,000 patients per year. The first year's sample is stratified by county and race/ethnicity (white vs. minority), and targeted at least 250 patients in each stratum with the remaining sample allocated proportional to county population size with oversampling of minorities. Patients who were alive, did not move between counties, and stayed enrolled in Medicare fee-for-service (FFS) were retained in the sample for subsequent years. Non-retained patients (those who died or were dropped from Medicare) were replaced with a sample of patients in their first year of Medicare FFS eligibility or patients who moved into a sampled county during the previous year.

**Results:**

The resulting sample contains an average of 899,266 ± 408 patients each year over the 10-year study period and closely matches population demographics and chronic conditions. For all years in the sample, the weighted average sample age and the population average age differ by <0.01 years; the proportion white is within 0.01%; and the proportion female is within 0.08%. Rates of 21 comorbidities estimated from the samples for all 10 years were within 0.12% of the population rates. Longitudinal cohorts based on samples also closely resembled the cohorts based on populations remaining after 5- and 10-year follow-up.

**Conclusions and relevance:**

This sampling strategy can be easily adapted to other projects that require random samples of Medicare beneficiaries or other national claims files for longitudinal follow-up with possible oversampling of sub-populations.

## Introduction

The United States (US) Medicare claims data capture national data on health care utilization for Americans aged 65 years old or older, disabled, or with end-stage renal disease (ESRD). Medicare currently is the only source of national data on healthcare utilization in the US, and thus its importance for epidemiological and health services research cannot be overemphasized (1–7). Due to the costs and sheer sizes of Medicare claims data, obtaining full data on even a subset (e.g., a disease-specific cohort) of the Medicare population with longitudinal follow-up over several years may not be feasible or practical. For this reason, researchers frequently work with a representative sample of the Medicare population.

Because the price for a Medicare claims file is the same for up to one million beneficiaries and increases thereafter, researchers tend to settle for a cohort with fewer than one million beneficiaries for each year. For projects with multiple objectives that require a longitudinal follow-up over many years as well as a cross-sectional analysis of a single year's data, however, this pricing structure creates a problem due to high attrition of Medicare population through mortality or disenrollment over time. In the example we discuss below, we observed that over 70% of patients in our sample exited Medicare fee for service (FFS) during a 10-year follow-up. Because of attrition and aging, the remaining cohort from the second year on will be substantially smaller than, and different from, the original cohort and older than the Medicare population overall for that year. Except for the first year, therefore, a longitudinal sample may not be representative of the Medicare population for subsequent years and cannot be validly used for population estimates.

On the other hand, if the data are sampled independently each year, the resulting data over multiple years may not be suitable for longitudinal analysis, because too few patients will be available for multi-year follow-up and the longitudinal sample would not be representative of the underlying longitudinal population due to independent sampling each year.

Therefore, a sampling approach suited for cross-sectional analysis may not be compatible with an approach optimized for longitudinal analysis. This poses dilemma for projects that require both longitudinal and cross-sectional analyses. This paper describes a method we developed for constructing a longitudinal sample from Medicare claims that is also valid for cross-sectional analysis. With this approach, researchers can maximize the value and utility of their Medicare samples. For small area variations between counties, our approach also takes oversampling into account for small counties and/or racial/ethnic minorities. To illustrate the method, we describe an ongoing research project studying Medicare patients with diabetes living in the Diabetes Belt and surrounding counties over a 10-year period (NIDDK R01DK113259). The sample for this project is also designed to be representative at the county level, and it additionally incorporates oversampling of whichever of the minority or white population of in each county is smaller.

## Methods

### Study sample, population, and data sources

Our study tracked Medicare patients with diabetes living in the area known as the Diabetes Belt (see below) and its surrounding counties between 2006 and 2015. Our initial cohort was a random sample of patients with diabetes identified from the 2006 Medicare records. These patients were then followed for 10 years until the end of 2015 to create a longitudinal sample. At the same time, some of our objectives required cross-sectional analysis (e.g., to examine risk factors for diabetic complications using the 2015 data), which necessitated careful augmentation of the data in each year of follow-up.

Our population included all Medicare Fee-for-Service patients with diabetes residing in the Diabetes Belt (described below) and surrounding counties. We used the Medicare Master Beneficiary Summary Files (MBSFs) to identify Medicare patients meeting inclusion criteria each year from 2006 to 2015. To be eligible for inclusion, Medicare patients needed to have been previously diagnosed with diabetes (identified in the Chronic Conditions segment in the MBSFs), be living in the Diabetes Belt or surrounding counties, and be enrolled in Medicare Fee-for-Service for all 12 months each year. Patients enrolled in Medicare HMOs were excluded because their claims data were not available.

### Diabetes belt and surrounding counties

The Center for Disease Prevention and Control (CDC) identified 644 counties across 15 states in the Appalachian region and the southeastern US as the Diabetes Belt (8). Some or all counties in Alabama, Arkansas, Florida, Georgia, Kentucky, Louisiana, Mississippi, North Carolina, Ohio, Pennsylvania, South Carolina, Tennessee, Texas, Virginia, and West Virginia comprise the Belt. We used the CDC's definition based on 2008 data in this study. We additionally identified 310 counties that are closest but not contiguous to the Belt counties as surrounding counties to serve as a basis for comparisons with the Belt counties. Counties that are immediately adjacent to the Belt were not included among the surrounding counties because some patients may cross county boundaries to seek care and may confound our estimates on healthcare utilization and outcome rates.

### Construction of the first year's sample

The sampling approach we developed was informed and influenced by our study objectives that included tracking changes in patient care, practice patterns, and outcomes over time. The sample we describe was designed to provide valid inference around these goals for patients with diabetes living in the Diabetes Belt and surrounding counties during the years from 2006 to 2015.

From a sampling design perspective, the goals we have outlined are somewhat in conflict. For example, if the goal is to provide similar precision within each county, then the optimal sampling design would be to sample approximately the same number of people in each county. In contrast, if the goal is to provide the best population-level estimates, then sampling from each county in proportion to its size is approximately optimal (9). The desire to compare white and minority populations in our study suggested oversampling of whichever group is smaller in each county. While surveys designed for a specific primary analysis can be further optimized, our survey needs to provide reasonable analytic power for multiple aims. This sampling design will provide good precision for a wide range of analyses.

Because the population sizes varied widely among counties, we chose to oversample patients from smaller counties to ensure they were adequately represented in our sample and to balance the competing needs for county and regional level inference. This meant that we must take the whole patient population from smaller counties. We thus allocated a minimum sample of 500 persons to each county or the county eligible population if < 500. We considered several alternatives between 500 and 1,000 and found that 500 allowed a complete enumeration for the smallest 18% of counties and at least 50% sampling for 70% of counties while still allowing significant sampling in the most populous regions. We then allocated the remaining available sample to each county proportional to the size of its un-sampled population, with the constant of proportionality chosen to produce a sample size as close as possible to the 900,000-person target; the resulting sampling rate was ~30% of the remaining population.

**Figure 1:**
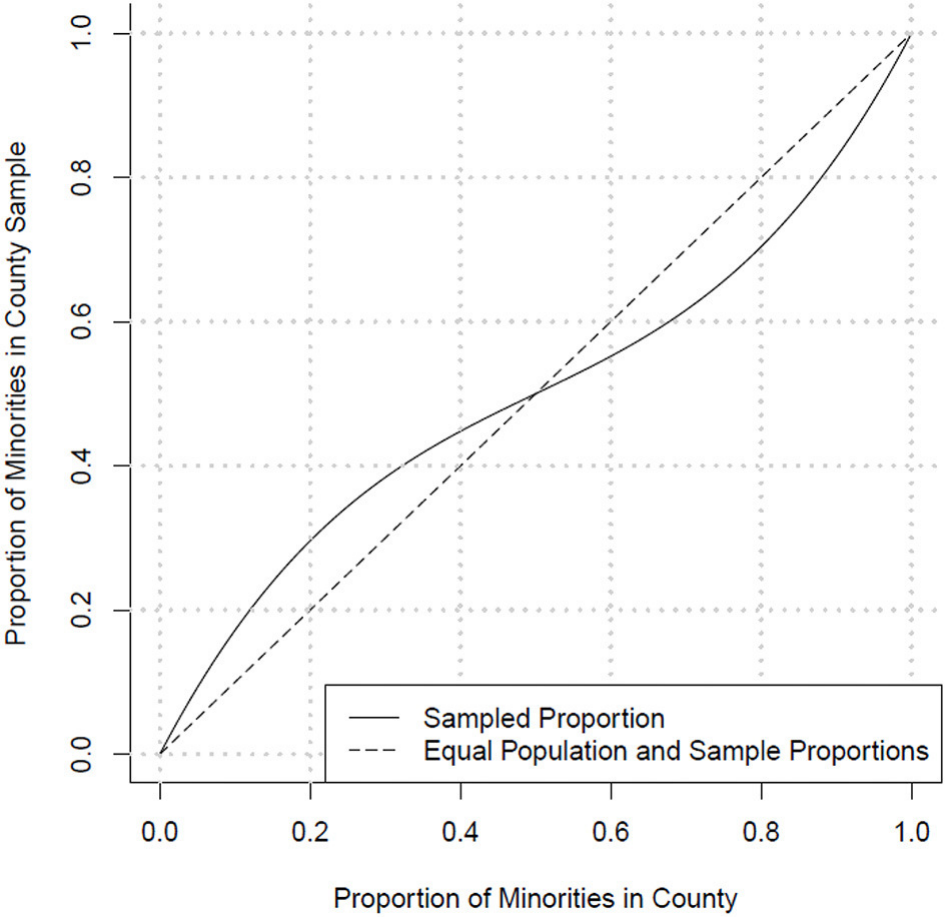
Targeted sampling proportion of minorities in each county.

Within counties we then initially tried to allocate a sample of 250 (or the population size if <250) to the white population and 250 for the minority population. Remaining samples allocated to the county were then divided between the white and minority populations according to the proportion.


$$\begin{array}{l} p_{ℑ}^{s}=2{\left(p_{ℑ}^{r}-\frac{1}{2}\right)}^{3}+\frac{\left(p_{ℑ}^{r}-\frac{1}{2}\right)}{2}+\frac{1}{2}, \end{array}$$

where $p_{ℑ}^{s}$ represents the minority proportion in the remaining sample for county *i*, $p_{ℑ}^{r}$ represents the minority proportion of the unsampled population of county *i* (Figure 1). In this paper, we combined all non-White racial/ethnic groups into one “minority” group. In counties where the minority population is smaller, this formula oversamples the minority population by a rate of approximately two-to-one when the minority population is proportionally small, transitioning to equal sampling as the white and minority populations become equal. In counties where the white population is smaller, the white population was oversampled. Our goal was to oversample whichever group (white/minority) was smaller in each county in order to improve within county comparisons while still providing significant coverage of the white population, which encompasses ~80% of the population living with diabetes in this region.

Once we had defined the sample size by stratum (county and white/minority), we then selected patients using simple random sampling within strata. Sampling weights were defined to be the stratum population size divided by the stratum sample size.

### Construction of subsequent years' samples

Sampling in subsequent years was complicated by the demands of retaining patients for longitudinal follow-up and ensuring a cross-sectionally representative sample in each year. Because the first year's sample (2006) was representative of all Medicare patients who had diabetes and met inclusion criteria in that year, all patients retained from the 2006 sample who had remained alive and eligible would have been representative of the population who had been eligible for at least 1 year (and they were therefore ~1 year older than the overall population). In order to replace patients in the 2006 data set who had died, enrolled in a Medicare HMO, or moved, we replaced them with an appropriately weighted sample of patients who became newly eligible for inclusion in 2007 by being new Medicare FFS enrollees or moving into a sampled county.

We constructed the 2007 replacement sample to first allocate at least 10 patients to each stratum to ensure that we add new beneficiaries in every county every year. Additional patients were then allocated to each stratum to target the overall sample size as would have been calculated using the 2006 sampling procedure on the 2007 county populations. All replacement patients were sampled from the population who would have been ineligible in 2006 (not enrolled in Medicare, in a Medicare HMO, lived elsewhere, or were first diagnosed with diabetes in 2007). Sampling weights were calculated as the number of first year eligible white/minority population in each county divided by the corresponding fill-in sample size. We similarly constructed the 2008–2015 replacement samples.

### Comparison of sample to population

In order to ensure the sample demographics reflected the underlying population for each year, we compared the randomly selected sample to the population. This analysis was performed using weighted survey sample analysis procedures (Stata “survey” suite of programs) with weights as described above and sampling strata defined by county, white/minority, and year the patient was added to the sample. Because the MBSF included limited number of patient characteristics, we only compared sample age, sex, race/ethnicity to the population parameters. Additionally, the Chronic Condition segment of the MBSF files contained indicators for 29 comorbid chronic conditions for each patient. We used them to compare the annual samples to the corresponding populations. We also constructed two longitudinal cohorts that were defined in 2006 and 2011 as the baseline (e.g., all patients with diabetes aged 65 years or older at baseline), respectively. We followed them for 10 and 5 years and patients remaining in the last year of follow-up (2015) were compared to the populations to examine how closely longitudinal cohorts constructed from samples resemble those constructed from the populations.

### Statistical analysis

Data cleaning was performed in SAS v9.4 (Cary, NC) and Stata SE v15.1 (College Station, TX); the random sample was generated using an R v3.6.1 (Vienna, Austria) program which is available on request. We compared the weighted sample values to the population parameters to test how closely our annual samples and longitudinal samples match annual and longitudinal populations. Descriptive statistics and comparison to the reference population were calculated using Stata survey programs. For standard statistics (means, proportions, totals, regression coefficients), Taylor-series-based methods were used for cross-sectional analyses presented below (10). This study was approved by the University of Virginia institutional review board.

## Results

Our study sampled Medicare FFS patients in each year of a 10-year period (2006–2015), retained as many patients as possible for longitudinal follow-up and allowed for cross-sectional analysis. We targeted a stratified random sample of about 900,000 from the Diabetes Belt and surrounding counties. Table 1 shows year-by-year retention based on year of initial sampling.

**Table 1:** Longitudinal retention (sample size and percent) in the Medicare data by year of initial inclusion and year of follow-up.

**Year of follow-up**	**Initial inclusion year**										**Total**
	**2006**	**2007**	**2008**	**2009**	**2010**	**2011**	**2012**	**2013**	**2014**	**2015**	
2006	899,846										899,846
	(100.0%)										
2007	758,145	140,283									898,428
	(84.3%)	(100.0%)									
2008	650,673	118,533	130,027								899,233
	(72.3%)	(84.5%)	(100.0%)								
2009	571,730	103,100	111,862	112,963							899,655
	(63.5%)	(73.5%)	(86.0%)	(100.0%)							
2010	498,506	90,072	96,198	95,862	118,543						899,181
	(55.4%)	(64.2%)	(74.0%)	(84.9%)	(100.0%)						
2011	442,871	80,911	85,636	83,960	102,075	104,002					899,455
	(49.2%)	(57.7%)	(65.9%)	(74.3%)	(86.1%)	(100.0%)					
2012	389,932	72,357	76,100	74,207	88,559	88,406	109,928				899,489
	(43.3%)	(51.6%)	(58.5%)	(65.7%)	(74.7%)	(85.0%)	(100.0%)				
2013	338,896	63,811	67,016	65,078	77,228	75,609	92,888	118,605			899,131
	(37.7%)	(45.5%)	(51.5%)	(57.6%)	(65.1%)	(72.7%)	(84.5%)	(100.0%)			
2014	291,437	56,067	58,805	57,016	67,392	65,440	79,394	99,827	123,466		898,844
	(32.4%)	(40.0%)	(45.2%)	(50.5%)	(56.9%)	(62.9%)	(72.2%)	(84.2%)	(100.0%)		
2015	252,870	49,980	52,255	50,724	59,695	57,696	69,796	86,294	104,600	115,491	899,401
	(28.1%)	(35.6%)	(40.2%)	(44.9%)	(50.4%)	(55.5%)	(63.5%)	(72.8%)	(84.7%)	(100.0%)	

Our sample design yielded an average sample size of 899,266 ± 408 over the 10-year study period. A total of 28% of the first year (2006) sample was retained for the full 10-year follow-up that included more than 200,000 non-Hispanic white and 70,000 minority patients. For a follow-up from 2010 to 2015 to examine the effects of the Affordable Care Act legislation, this sampling approach yielded a sample of over 460,000 patients (52% of the 2010 sample) who can be followed up for the full 6 years. Although Hispanic and other race/ethnicity groups represented <1% of our total sample, the study retained a substantial number (~2,000 or more) for area-wide subgroup comparison and for longitudinal follow-up for the 10-year period.

In order to assess the resulting sample, for each year of the survey we made cross-sectional comparisons of the weighted sample to the population defined from the MBSF (described in Table 1). Population size, race, and previous year sample eligibility were the factors we used in determining the sample. We therefore focused our descriptive statistics on race, age, sex, and population totals. Age is a particularly important variable for assessment because if the fill-in samples were incorrectly constructed, we would expect to see drift from the underlying population as the retained samples from previous years aged. We additionally included sex because it is an important factor in most health outcomes and it provides a good additional point of comparison that was not incorporated in the sampling design.

**Figure 2:**
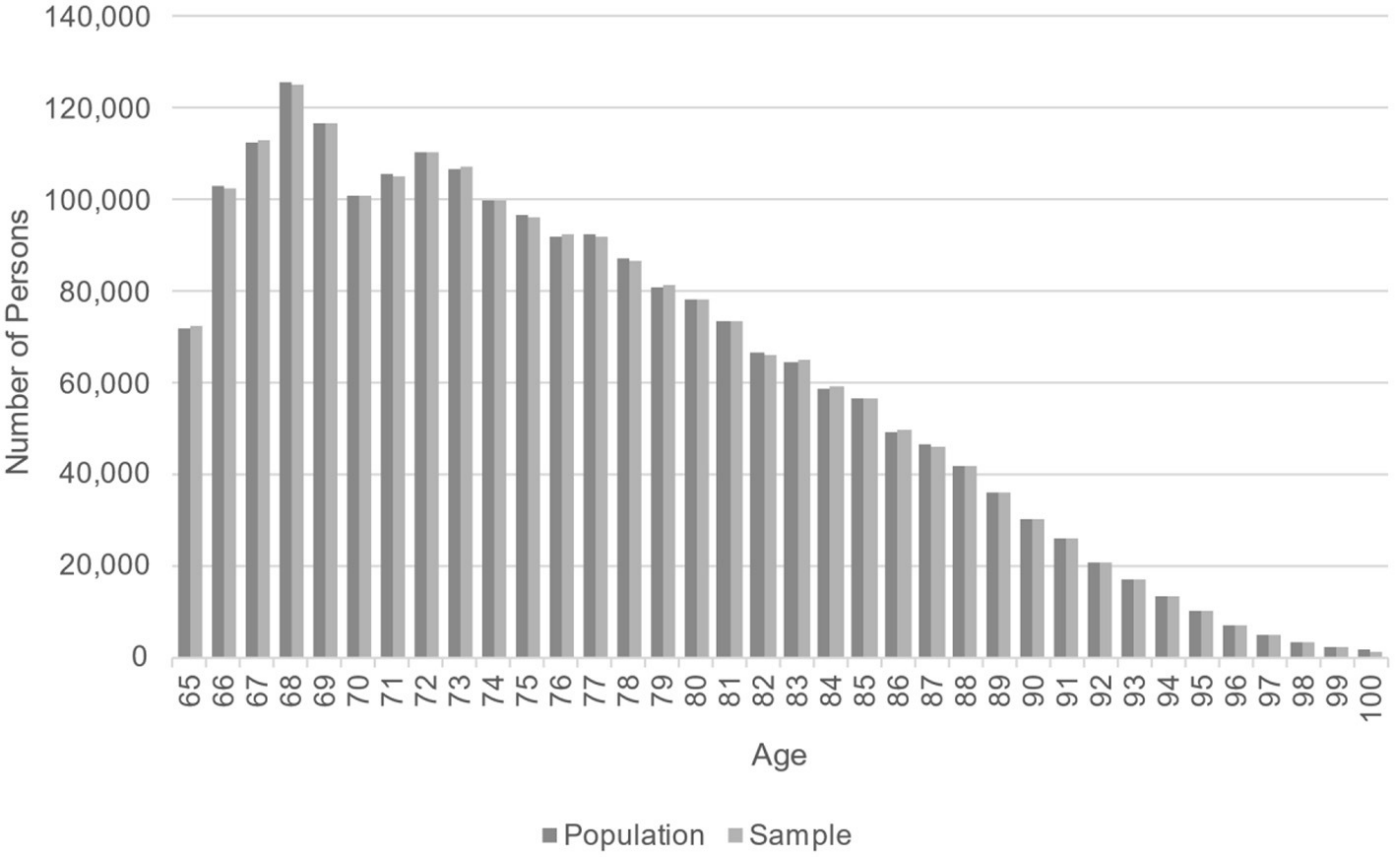
Comparison of population and weighted sample age distribution for 2015.

Demographic comparisons are shown in Table 2. For all years in the sample, the weighted sample average age and the population age differ by < 0.01 years; the proportion white is within 0.01%; and the proportion female is within 0.08%. No difference was statistically significant at the α = 0.05 level. Figure 2 shows that, in the last year of follow-up (2015), the weighted age distribution in the sample closely matched the population age distribution. This comparison provides a visual check that the fill-in samples from years 2007–2015 were appropriately weighted to allow for valid cross-sectional comparisons of age.

**Table 2:** Cross-sectional comparison of population and weighted sample by age, sex, and race/ethnicity by year.

**Year**	**Age**			**Sex (% male)**			**% Non-Hispanic white (or missing)**		
	**Population**	**Sample**	***p*-value**	**Population**	**Sample**	***p*-value**	**Population**	**Sample**	***p*-value**
2006	75.09	75.09	0.927	818,368 (42.9%)	812,114 (42.8%)	0.193	1,494,096 (78.8%)	1,494,096 (78.8%)	1.000
2007	75.19	75.19	0.896	828,638 (43.2%)	826,912 (43.2%)	0.126	1,524,182 (79.5%)	1,523,807 (79.5%)	0.930
2008	75.24	75.23	0.949	844,511 (43.4%)	842,987 (43.4%)	0.249	1,543,822 (79.4%)	1,543,209 (79.4%)	0.888
2009	75.29	75.29	0.718	867,876 (43.6%)	866,994 (43.5%)	0.497	1,573,679 (79.0%)	1,573,056 (79.0%)	0.764
2010	75.37	75.37	0.871	890,438 (43.8%)	890,163 (43.8%)	0.872	1,599,612 (78.6%)	1,598,752 (78.6%)	0.927
2011	75.38	75.38	0.641	929,910 (44.0%)	930,815 (44.0%)	0.361	1,658,040 (78.4%)	1,657,943 (78.4%)	0.956
2012	75.32	75.33	0.317	955,839 (44.2%)	956,853 (44.2%)	0.261	1,692,521 (78.2%)	1,692,285 (78.2%)	0.979
2013	75.30	75.31	0.267	970,658 (44.5%)	971,854 (44.5%)	0.255	1,702,352 (78.0%)	1,702,401 (78.0%)	0.975
2014	75.27	75.27	0.860	978503 (44.8%)	980,321 (44.9%)	0.117	1,700,496 (77.9%)	1,700,924 (77.9%)	0.909
2015	75.27	75.27	0.958	997,913 (45.1%)	999,868 (45.2%)	0.170	1,717,930 (77.6%)	1,719,008 (77.6%)	0.834

At the county level, the sample produced a complete census for at least 14.5% of counties each year, and 58.6% counties had more than 50% of their residents capture each year. All counties had more than 25% of their residents captured every year.

Prevalence rates of 21 chronic conditions were compared between the population and the sample using one-sample proportion test. Table 3 shows the comparisons for 3 years only (2006, 2010, and 2015). Overall, we found that rates estimated from the samples for all 10 years closely approximated the population parameters; they were within 0.12% of the population rates. However, we found that five of the 210 comparisons (0.24%) showed statistically significant differences from the underlying population. We found no apparent trend or pattern for the bias over time. Some uncommon conditions such as endometrial cancer at around 1% were well-represented in the samples over the study period.

**Table 3:** Comparison of prevalence rates of 21 chronic conditions from the population and the weighted samples for 2006, 2010, 2015.

**Conditions**	**2006**			**2010**			**2015**		
	**Pop**	**Sample**	***P*-value**	**Pop**	**Sample**	***P*-value**	**Pop**	**Sample**	***P*-value**
AMI	7.48%	7.48%	0.978	8.08%	8.10%	0.529	7.99%	8.07%	0.022
Atrial fibrillation	17.88%	17.90%	0.596	19.03%	18.99%	0.425	20.02%	19.97%	0.230
CKD	26.07%	26.09%	0.655	34.43%	34.46%	0.596	44.17%	44.20%	0.607
COPD	31.76%	31.69%	0.155	33.89%	33.85%	0.490	34.13%	34.12%	0.750
CHF	41.47%	41.45%	0.765	41.06%	41.01%	0.415	38.12%	38.10%	0.739
Hip fracture	4.42%	4.43%	0.456	4.89%	4.89%	0.741	4.74%	4.73%	0.723
IHD	60.06%	60.01%	0.453	61.96%	62.02%	0.271	60.09%	60.10%	0.729
Depression	26.91%	26.98%	0.187	32.07%	32.09%	0.730	37.42%	37.37%	0.391
RA/OA	52.16%	52.19%	0.653	59.24%	59.18%	0.243	63.86%	63.84%	0.731
Stroke	21.03%	21.13%	0.039	21.90%	21.91%	0.818	21.35%	21.40%	0.346
Breast cancer	4.83%	4.82%	0.494	5.35%	5.34%	0.792	5.79%	5.77%	0.441
Prostate cancer	5.64%	5.64%	0.793	6.16%	6.17%	0.736	6.31%	6.34%	0.225
Colorectal cancer	3.67%	3.67%	0.747	3.70%	3.71%	0.463	3.54%	3.55%	0.630
Lung cancer	1.84%	1.81%	0.065	1.97%	1.95%	0.189	2.01%	1.98%	0.089
Endometrial cancer	0.80%	0.79%	0.716	0.87%	0.87%	0.391	1.07%	1.06%	0.336
Anemia	55.88%	55.84%	0.506	62.04%	61.98%	0.298	63.76%	63.75%	0.751
Asthma	12.09%	12.06%	0.414	14.63%	14.61%	0.512	16.62%	16.62%	0.993
Hyperlipidemia	79.90%	79.89%	0.872	88.37%	88.38%	0.893	91.63%	91.65%	0.500
BPH	16.50%	16.49%	0.801	19.39%	19.34%	0.312	21.56%	21.58%	0.716
Hypertension	92.94%	92.92%	0.538	95.13%	95.10%	0.377	95.53%	95.54%	0.702
Hypothyroidism	22.32%	22.29%	0.419	26.53%	26.47%	0.276	30.47%	30.44%	0.601

AMI, acute myocardial infarction; CKD, chronic kidney disease; COPD, chronic obstructive pulmonary disease; CHF, congestive heart failure; IHD, ischemic heart disease; RA/OA, rheumatoid arthritis/osteoarthritis; BPH, benign prostate hyperplasia.

Comparison of longitudinal cohorts from samples resembled the matching cohorts assembled from the populations in the last year of follow-up (Table 4). For this comparison, we removed a small percentage of patients who moved out of a county during a 10-year (3.2%) and 5-year (1.8%) follow-up. We found that the sample and population retained 30 and 31% of those in the baseline year at the end of the 10-year follow-up and 60 and 61% after the 5-year follow-up. Average ages in the remaining cohorts differed by 0.01 and 0.003 years, respectively, after a 10- and 5-year follow-up between sample and population cohorts. Gender and race/ethnicity distributions in the sample cohorts were within 0.2% of the population cohorts. Similarly, comorbidity distributions were also very similar after 10- and 5-year follow-up (≤0.2% for all conditions). Only four of 48 comparisons (8%) showed statistically significant differences between population and sample values in Table 4.

**Table 4:** Longitudinal cohorts from annual samples compared to matching cohorts from populations^*^.

**Variables**	**10-year follow-up**				**5-year follow-up**			
	**Pop**	**Sample**	**Diff**	***P*-value**	**Pop**	**Sample**	**Diff**	***P*-value**
Cohort retained last year	31.0%	30.8%			61.4%	60.0%		
Age in 2015 (mean)	82.1	82.1	0.01	0.299	79.0	79.0	0.00	0.764
Male	42.9%	43.0%	−0.13%	0.212	44.1%	44.3%	−0.16%	0.037
NH White	80.2%	80.2%	−0.03%	0.661	78.9%	78.9%	−0.02%	0.038
AMI	11.2%	11.2%	0.00%	0.951	9.4%	9.5%	−0.08%	0.070
Atrial fibrillation	27.1%	27.0%	0.03%	0.734	23.1%	23.1%	0.05%	0.465
CKD	55.3%	55.4%	−0.03%	0.747	49.2%	49.2%	−0.07%	0.342
COPD	39.4%	39.2%	0.21%	0.040	37.1%	37.1%	0.01%	0.876
CHF	49.9%	49.9%	0.09%	0.420	43.4%	43.4%	0.02%	0.831
Hip fracture	7.2%	7.2%	0.06%	0.245	5.6%	5.6%	−0.01%	0.788
IHD	72.4%	72.4%	0.04%	0.686	66.6%	66.6%	−0.06%	0.386
Depression	39.3%	39.1%	0.18%	0.088	38.5%	38.4%	0.09%	0.202
RA/OA	75.8%	75.6%	0.19%	0.039	71.0%	71.0%	0.05%	0.497
Stroke	28.3%	28.4%	−0.11%	0.242	24.4%	24.5%	−0.08%	0.206
Breast cancer	7.1%	7.1%	0.05%	0.335	6.5%	6.4%	0.04%	0.254
Prostate cancer	8.1%	8.1%	−0.02%	0.762	7.3%	7.3%	−0.04%	0.260
Colorectal cancer	4.9%	4.9%	0.01%	0.784	4.2%	4.2%	−0.01%	0.641
Lung cancer	2.2%	2.2%	0.05%	0.104	2.1%	2.1%	0.02%	0.350
Endometrial cancer	1.3%	1.3%	0.01%	0.734	1.2%	1.2%	0.02%	0.264
Anemia	77.7%	77.6%	0.09%	0.291	71.1%	71.1%	0.02%	0.750
Asthma	18.1%	18.0%	0.04%	0.632	17.8%	17.8%	−0.01%	0.925
Hyperlipidemia	96.3%	96.3%	−0.01%	0.842	95.2%	95.2%	−0.02%	0.580
BPH	27.2%	27.2%	−0.02%	0.825	24.7%	24.7%	0.00%	0.966
Hypertension	98.6%	98.6%	0.02%	0.420	97.7%	97.7%	0.03%	0.291
Hypothyroidism	36.1%	36.0%	0.12%	0.253	33.4%	33.3%	0.08%	0.278

^*^Data show comparisons for patients retain in the last year of follow-up (2015). The 10-year and 5-year follow-up started in 2006 and 2011, respectively. AMI, acute myocardial infarction; CKD, chronic kidney disease; COPD, chronic obstructive pulmonary disease; CHF, congestive heart failure; IHD, ischemic heart disease; RA/OA, rheumatoid arthritis/osteoarthritis; BPH, benign prostate hyperplasia.

## Discussion

In this paper, we described a sampling method which produced a representative sample of the US Medicare patients for a 10-year study period. This method produced annual samples that closely matched the populations in demographics and comorbidity distributions. The longitudinal cohorts defined using annual samples also closely resembled those defined using annual populations in the percentages of retained patients, demographics, and comorbidity rates in the last year of 10- and 5-year follow-up.

Our results show that the properly weighted sample and the population had almost identical age and sex and race distribution each year over the 10-year follow-up. Unlike a pure longitudinal sample for a retrospective cohort study, the sample for each year is a good representative sample that has virtually identical distribution of age, sex, race/ethnicity, and comorbid conditions to the population not just in the baseline year but also in all subsequent years.

We also showed that disease prevalence rates in the sample closely approximated the rates from the population; all differences were ≤ 0.12% of the population rates. The five of 210 comparisons (0.24%) that showed significant differences from the underlying population; this however is less than the alpha = 5% Type I error rate that would be expected from a perfectly representative sample. The samples also performed well in representing beneficiaries with uncommon conditions such as endometrial cancer. This suggests that the annual samples taken using our proposed method can be used to estimate rates of medical conditions in the population with reasonably high accuracy.

Retrospective cohorts defined using samples and populations were remarkably similar even after a long follow-up. Our data also suggest that retrospective cohorts can be defined in the middle of a study period and the resulting longitudinal sample and follow-up data will closely resemble those in the matching population.

A primary objective in this work was to document our sampling approach for future researchers who might be interested in obtaining representative samples of Medicare claims data. In preparing for this project, we found only limited literature describing longitudinal sampling designs that could serve as a reference. Smith et al. (11) offers a very high-level overview and describes the principles of sampling design for longitudinal surveys. Other articles address subsets of our challenge. For example, Wolinsky et al. (12) discusses matching Medicare claims to a longitudinally followed cohort without need for cross-sectional inference, while Thompson (13) and Carrillo and Karr (14) focus primarily on analytic approaches rather than design.

In constructing this sample, we found that it was relatively easy to produce a representative sample for the baseline year (2006), even with stratification and oversampling. Significantly more care was needed to identify the sampling frame for subsequent years. An advantage of working with Medicare data and with samples this large is that there is abundant power to identify potential problems before purchasing data.

The samples derived using our approach can be flexibly used for both longitudinal and cross-sectional analysis, trend analysis, and other studies that require more complex designs such as nested case-control designs or interrupted time-series designs. As an illustration, this sampling approach produced yearly samples of US Medicare beneficiaries with diabetes residing in the Diabetes Belt and surrounding counties that we used to assess trends in preventive care utilization, long-term outcomes, disparities, and associations between preventive care and diabetic complications in patients with diabetes. These goals required the sample to be valid both longitudinally and cross-sectionally. Because we took the population (e.g., all eligible beneficiaries) from small counties, these samples can provide as much power to make county-level comparisons as the population does.

This study is limited by the scope of Medicare claims data. In particular, this cohort only included patients ages 65 and up, and it excluded those patients enrolled in a Medicare HMO. In addition, Medicare only captures claims data, so we did not have access to full clinical records.

## Conclusions

We demonstrated that a representative sample of Medicare beneficiaries can be carefully constructed to be used in cross-sectional as well as longitudinal analyses. This sampling method makes the data request much more affordable. The computer algorithms we created can be used by future researchers in drawing random representative samples from Medicare claims data.

## Data availability statement

The data analyzed in this study is subject to the following licenses/restrictions: the data used in this study was obtained under a data use agreement with the US Centers for Medicare and Medicaid Services that restrict sharing of data. Requests to access these datasets should be directed to JL, jem4yb@virginia.edu.

## Ethics statement

The studies involving humans were approved by the University of Virginia Institutional Review Board for Health Sciences Research. The studies were conducted in accordance with the local legislation and institutional requirements. Written informed consent for participation was not required from the participants or the participants' legal guardians/next of kin in accordance with the national legislation and institutional requirements.

## Author contributions

TM: Conceptualization, Formal analysis, Methodology, Software, Validation, Visualization, Writing – original draft, Writing – review & editing. JL: Data curation, Funding acquisition, Project administration, Supervision, Writing – review & editing. SK: Formal analysis, Validation, Writing – review & editing. HK: Writing – review & editing. M-WS: Conceptualization, Data curation, Formal analysis, Funding acquisition, Methodology, Project administration, Supervision, Validation, Writing – original draft, Writing – review & editing.

## Appendix A

### Example sample construction and weight calculation

As an example of how the initial and fill-in samples were constructed, consider a hypothetical county with 2006 population of 1,600 white and 400 minority eligible Medicare patients with diabetes and a target sampling rate of 50% (see Construction of first year's sample). Initially, 250 samples are allocated to the white population and 250 to the minority, leaving 500 additional samples to be allocated across 1500 remaining patients. Ten percent (150) of the remaining patients are minority, so we would target the remaining sample to be 17.2% minority (26 patients). Therefore, the 2006 sample would contain 276 minority patients and 724 white patients, and the sampling weights for the minority patients will be 1.45 and the sampling weights for white patients would be 2.21.

In 2007, imagine that of the 2006 sample, 615 white and 235 minority patients remained enrolled in Medicare and living in county in 2007. These 850 patients would be retained for 2007 with their 2006 sampling weights. The 150 sampled patients who were lost would be replaced from newly eligible Medicare enrollees. Imagine that there were 300 newly eligible enrollees in our county in 2007, 260 white and 40 minority. We would immediately allocate 10 samples to the white stratum and 10 to the minority. For the remaining 130 samples, we target 18.2% minority (51) patients, but since only 40 minority patients are available, the 2007 fill-in sample will be 40 minority patients each with sampling weight 1, and 110 white patients each with sampling weight 2.36.

Samples in years 2008 and on would be constructed similarly to the 2007 sample. An R program for selecting samples and computing weights is available on request.
